# Informal carers in Sweden – striving for partnership

**DOI:** 10.1080/17482631.2021.1994804

**Published:** 2021-10-31

**Authors:** Elin Blanck, Andreas Fors, Lilas Ali, Margareta Brännström, Inger Ekman

**Affiliations:** a Institute of Health and Care Sciences, Sahlgrenska Academy, University of Gothenburg, Gothenburg, Sweden; b Centre for Person-Centred Care (GPCC), University of Gothenburg, Gothenburg, Sweden; c Research and Development Primary Health Care, Gothenburg, Sweden; d Psychiatric Department, Sahlgrenska University Hospital, Gothenburg, Sweden; e Department of Nursing, Umeå University, Campus Skellefteå, Sweden

**Keywords:** Carers, chronic illness, chronic obstructive pulmonary disease, heart failure, qualitative research, support

## Abstract

**Purpose:**

Informal carers have an important role in society through their care and support of their long-term ill relatives. Providing informal care is challenging and can lead to caregiver burden; moreover, many support needs of the carers are not met, leading to confusion, disappointment and frustration. We conducted an interview study to clarify the meaning of support given and received by informal carers to relatives with chronic obstructive pulmonary disease or chronic heart failure.

**Methods:**

We purposively selected and recruited informants via participants in another study, thereby conducting interviews over the phone from June 2016 to May 2017. In total, we conducted 14 interviews with 12 informants. All interviews were transcribed verbatim and the content was analysed using a phenomenological hermeneutical approach.

**Result and conclusion:**

Our comprehensive understanding of the meaning of support for these carers is twofold: it is a self-evident struggle for the good life of their relatives and that they want to be *carers in partnership*. The healthcare system must recognize the efforts of carers and include them in the strategic planning and operational stages of care and treatment for people with long-term illness.

## Introduction

The support that family or friends give to people living with a long-term illness is of paramount importance. Informal carers play a significant role in supporting patients with chronic obstructive pulmonary disease (COPD) or chronic heart failure (CHF) in their self-care capability (Bryant et al., 2016; Buck et al., 2018; Kitko et al., 2020). An informal carer can be a relative, a neighbour or a friend. In this paper the term carer describes a person providing care in an unofficial capacity. In Europe, surveys show that one in three people provide care to a closely connected person (i.e., family members and persons with whom you have a close association) who has a long-term illness, disability or problems associated with old age. In Sweden, where this study was conducted, it is estimated that almost 40% of those above 18 years provides help, support and care on a regular basis (Verbakel et al., 2017). Swedish law and recommendations says that these informal carers are to be supported in their turn by the municipality they live in; this support can be for example, support and information groups, home-care or assistance or respite care (Eurocarers, 2021). Verbakel et al. (2017) also describe the prevalence of what they define as intensive caregiving, that one spends 11 hours or more per week on caregiving, and they estimates that 4.5% of those 25 years or older belongs to this category in Sweden.

For patients with CHF, carers seem to positively influence outcomes such as re-hospitalization and mortality (Luttik et al., 2005). Similarly, carers positively impact smoking cessation and adherence to prescribed medications in patients with COPD (Trivedi et al., 2012). Many COPD and CHF symptoms are similar: both are characterized by fatigue and shortness of breath (Theander et al., 2014), and the conditions are common. COPD represents the fourth leading cause of death globally and is estimated to become the third most common cause of death by 2020 and it has an estimated global prevalence of 11.7% (GOLD, 2020). In Sweden the prevalence of COPD is estimated to be 7.0% (Backman et al., 2020). CHF has a prevalence of up to 10% in people >70 years (Ponikowski et al., 2016) and in Sweden, its prevalence in the whole population is estimated to be 2% (Zarrinkoub et al., 2013) with more than 90% of persons with CHF being 60 years or older. The reoccurring exacerbations of COPD lead to disturbed sleep, decreased physical activity and an increased risk of depression and anxiety (Miravitlles & Ribera, 2017). Living with CHF, often resulting in social isolation, fear and a loss of control (Jeon et al., 2010), has been described as “feeling imprisoned in illness” (Ekman et al., 2000).

Planning activities for people with COPD and CHF is difficult, partly because of their shifting energy levels. Many of these people require support from their next of kin and society (Johansson et al., 2019; Kitko et al., 2020). The presence of social support, defined as living with others and having a carer, is associated with enhanced health-related quality of life (HRQoL) and higher levels of self-care, including physical activity or participation in rehabilitation programmes (Chen et al., 2017) (Årestedt et al., 2013). Iqbal et al. found that lack of social support for patients with CHF results in lower HRQoL apart from more hospital admissions and death (Iqbal et al., 2010).

Simultaneously, the informal care of people with chronic conditions has been linked to caregiver burden (Burton et al., 2012; Garlo et al., 2010; Strömberg, 2013). Studies indicate that carers have an increased risk of developing depression and are less likely to seek health care for themselves (Badr et al., 2017; Berglund et al., 2019).

There is a general lack of knowledge concerning the needs of carers (Caress et al., 2009) and a recent review (Micklewright & Farquhar, 2020) concludes that many of the support needs of carers may go unmet.

Moreover, as chronic conditions increase, the contributions of carers are also likely to increase. Health care faces challenges in finding ways to include carers in the strategic planning and operational stages of care tasks and supporting them in their caregiving role. Consequently, more knowledge is needed about what it means for carers to support others and how they experience their situation (i.e., when they act as support for their relatives). Therefore, this study aimed to elucidate the meaning of support given and received by carers of close relatives with COPD or CHF.

## Methods

### Design

We conducted an interview study with a qualitative, descriptive design using a phenomenological hermeneutical approach to interpret the interview text.

### Recruitment of informal carers

The informants were recruited through a cohort of participants enrolled in a previous trial (Fors et al., 2018) evaluating the effects of person-centred phone support in patients with COPD, CHF, or both. These patients were included after being admitted to hospital because of their worsened pre-existing clinical condition. The study was conducted in the west of Sweden, in a mostly urban area. We used a purposive sampling strategy by asking the participants in the intervention group who had received more than one support phone call if they had an carer who might be willing to participate in an interview study. At the time of inclusion for this study, 58 of the participants in the intervention group were possible to ask, and out of these we were able to contact 25 as some of the included patients had died or were admitted to hospital or otherwise not possible to reach. After we collected a list of potential participants, we phoned to inform them about the study and asked if they would be willing to participate. If they agreed to participate in the study, we mailed each prospective participant written information about the study and an informed consent form to be returned by post. If they returned a signed informed consent form, we contacted them (by phone) once again to schedule an interview. Eleven persons out of 25 said that they did not think that their relatives would have something to add, and one relative declined participation for that same reason. One relative that agreed to participate did not return his signed consent form and was therefore excluded. Informant characteristics are described in Table I.

**Table I.** Overview of the informants and their relatives

Informant no.	Age	Relation	Condition of relative	Interview no.
1	60	Daughter	COPD^1^	1 + 14
2	65	Daughter	CHF^2^	2
3	64	Wife	CHF	3
4	66	Wife	CHF	4 + 6
5	72	Niece	CHF	5
6	62	Daughter	COPD	7
7	53	Daughter	COPD	8
8	60	Wife	COPD	9
9	44	Daughter	COPD	10
10	77	Wife	CHF	11
11	65	Wife	CHF	12
12	72	Wife	CHF	13

### Interviews

All interviews were conducted by phone between June 2016 and May 2017. In all, 14 interviews were performed with 12 persons (two of the informants were interviewed twice). During the first four interviews, some focus was on the phone support that the informants’ relatives had received during the intervention. However, we realized that it was hard for informants to separate their experience of the support their relatives received through the intervention from other support received and, we wanted to deepen our understanding of what support could mean for the carers, so attempts were made to re-interview the first four informants. However, only two of these four informants responded and agreed to a second interview. The informants had different family ties to the person they cared for (six wives, five daughters and one niece). The interviews ranged from 10 to 48 minutes (mean 25 minutes). The first author conducted nine interviews and the fourth five interviews. The first author is a female RN and a PhD student with limited experience of interviewing, but she was supervised in between the interviews and discussed them with the co-authors who are more experienced. The fourth author is a female RNT and professor, and she has extensive experience in interviewing. The interviews were semi-structured, consisting of open-ended questions. Could you tell me what support in your role as a carer means to you? Can you give me an example of when you were supported in caring for your relative? For additional questions asked during the interviews, please see the interview guide (Supplementary file 2).

### Analysis

The interviews were transcribed verbatim by the first and fourth author and then analysed using a phenomenological hermeneutical method inspired by Ricœur (1976) and developed for research by Lindseth and Norberg (2004). This method, which can be described in three interrelated steps, has been used in numerous studies (e.g., Brännström et al. (2007) and Ali et al. (2018)):

- Repeated reading of the text was conducted to acquire a naïve understanding of the content.
- Structural analysis was performed in which the text is divided into meaning units that are condensed to form the themes and sub-themes.
- Preunderstanding, naïve understanding and structural analyses are weighed together to create a comprehensive understanding of the studied phenomena.

In practice, a phenomenological hermeneutical analysis moves between understanding, explaining and interpreting the whole and parts of the text. The complete transcribed interviews were read repeatedly, and a naïve understanding was formulated and discussed among the authors. The naïve understanding is a first impression concerning the meaning of the text, an idea about what the text says. After that, the text was re-read and divided into meaning units, which were condensed and read again. The condensed meaning units were sorted together with similar ones to make up sub-themes of the structural analysis. These sub-themes were then grouped to form the core themes. In this study the structural analysis was mainly performed by the first author, but all authors engaged in the process. An example of the process of moving from a meaning unit to a theme is given in Table II.

**Table II.** An overview of the structural analysis process

Meaning unit	Condensation	Sub-theme	Theme
She (her mother) has continued coming to the lung clinic at the local hospital, and it has been great. They are great there. I can call them and ask what do we do now? Or what do I do now? Because I have missed that in healthcare. Someone to turn to instead of the emergency room or the big hospitals. (Informant 1)	They are great at the local clinic. I can call them and ask what to do. I have missed having someone to turn to within healthcare.	Being met with respect	Experiencing the unpredictable healthcare system
Mostly things have been very good at the primary healthcare centre, and my husband has a very accommodating and very good doctor. And some of the nurses there as well. When we were on vacation in Spain, my husband became ill with his blood pressure, and instead of seeking care urgently, I could call home to his reception, and then they called back and helped so that we could get guidance in changing the medication. They called back and helped, so that was absolutely incredible! (Informant 12)	My husband’s doctor and some of the nurses have been very accommodating. When my husband became ill with his blood pressure while on vacation, I could call, and they called back and guided us in changing the medication. That was incredible!		

Finally, the naïve understanding and the themes were interpreted as a whole and reflected upon from the authors’ pre-understandings or presuppositions and research related to the area to form a comprehensive understanding of the studied phenomena.

The consolidated criteria for reporting qualitative research (COREQ) checklist (Tong et al., 2007) was applied as the reporting guideline for this study (Supplementary File 1).

### Ethical approval

The study was approved by the Regional Ethics Board (Ref no. 687-14 and ref no. T129-16) and conformed with the principles outlined in the Declaration of Helsinki (World Medical Association, 2013).

## Findings

The findings are presented in three parts: 1) naïve understanding, 2) themes of the structural analysis and 3) comprehensive understanding.

### Naïve understanding

The naïve understanding of what support can mean for someone caring for a relative with COPD or CHF is that support is given wholeheartedly and with deep conviction. The carers are significant and important persons in the life of their relatives. However, it can also be frustrating for the carers when they cannot influence decisions or acquire information from (or contact) the “right” persons (within the healthcare system).

### Structural analysis

**Figure 1.**
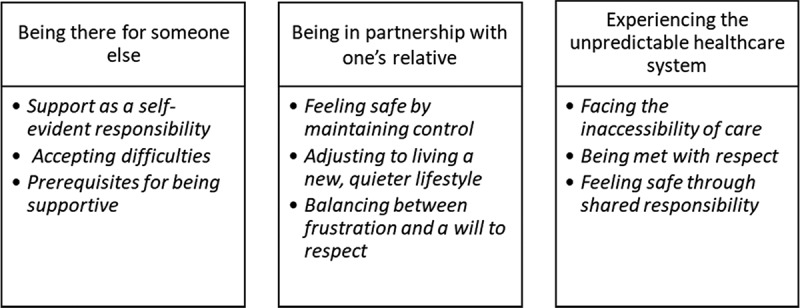
Overview of the strucural analysis: Themes and associated sub-themes

The themes and associated sub-themes are described below and depicted in Figure 1.

### Being there for someone else

#### Support as a self-evident responsibility

The carers were present in everyday events to support their relatives. Spouse carers noted that the relationship with their partner was an advantage as years of marriage made it possible to anticipate their partner’s needs.

To be there, to always walk beside him- that is support to me. (Informant 12)

The carers spent considerable time and effort providing practical and emotional support to their relatives. Moreover, they often had daily contact despite not living with the relative or nearby. They saw their support as a natural and self-evident activity of everyday life. They wanted to comfort their relatives, especially if the relatives’ situation worsened because they did not see anyone else in a similar position to help.

#### Facing difficulties

Although providing support was an obvious response, it could also be challenging to witness the suffering of loved ones.

Sometimes I have lost my temper, it has been hard to see him lose weight, he barley ate at all and became thinner and thinner. I tried to cook different meals, but nothing tasted well. Therefore, I got mad sometimes, you will never make it through a heart transplant if you do not eat, recover and get stronger. (Informant 3)

Carers often became frustrated by the difficulties that arose when coordinating with healthcare. Some questioned whether they had the strength to continue. Moreover, the emotional stress of providing care and support was intensified, knowing that their relative’s condition would only worsen, resulting in progressive suffering and eventual death. Although the carers knew the outcome of the disease progression, they were not completely prepared for such a tragic loss.

#### Prerequisites for being supportive

For many carers, their circumstances made it possible to provide support, including having a job that allowed a flexible work schedule, giving them a level of autonomy. Several of the carers reported yearning for the opportunity to take time off from work to help a sick relative. Maintaining an encouraging and cheerful outlook was also construed as a prerequisite for good care. The families and friends of many carers gave support by showing understanding and allowing the carers an opportunity to express their concerns and worries. Sometimes the carers received help in unexpected ways, which was a great source of inspiration.

One day when I came home from work, and my husband was at home sick … he had not noticed anything … our neighbours had been here and shovelled our entire long path free from snow; they just came over and did it. (Informant 3)

### Being in partnership with one’s relative

#### Feeling safe by maintaining control

Because carers often were responsible for monitoring their relative’s condition, they were required to keep close contact, even though they did not live with their sick relative. The sick relative would contact the carer if his or hers condition worsened and the carer was then responsible for contacting and coordinating with the healthcare system.

The home care service usually calls for the ambulance if she gets ill, but I drive her to the hospital by myself regardless of how ill she is. Maybe that is somewhat stupid of me, but I prefer to keep some kind of control myself. (Informant 10)

Attending their relative’s appointments and care planning meetings felt important because the carer wanted to help understand and remember the healthcare professionals’ information. In addition, during these meetings, the carers often served as the relatives’ voice, advocating for their needs and wants. After these meetings, the carers would often research the information discussed (e.g., read about the adverse effects of medication).

#### Adjusting to living a new, quieter lifestyle

The carers had to adjust their lifestyle to the needs of their sick relatives.

Therefore, we cannot travel the way we had hoped. We were told it is best not to, so we have a slow lifestyle, as he does not have the energy for long walks. Therefore, they have to be shorter. Somehow, we find ways to adapt, but it is mostly I who must adapt. Actually, that is the way it is; of course, it would be strange otherwise … this is what is required of a relative, to adapt. (Informant 8)

Activities or social events that the sick relative would not have the energy to participate in were avoided. The carers cancelled or postponed such activities if the condition of their loved ones required it. At times, they felt that they could not relax or leave their relatives alone, which could be incredibly stressful. Not being able to travel or do other things they normally do could lead to a more modest and reserved lifestyle. Some carers described that they sometimes had the sole responsibility for some actions they earlier shared with their partners (e.g., practical chores or decisions about the economy).

#### Balancing between frustration and a will to respect

This sub-theme was about to what extent the carers could influence their relatives and their decisions. They did not want to dominate their loved ones. Rather, they preferred to show respect for the relatives’ autonomy, but at the same time, it was frustrating knowing that the relatives’ life could have been better if they had moved to another place or made better health choices.

But it would have been a dream if they had moved 30 years ago to a home for seniors. Now she is a prisoner in her apartment. (Informant 5)

Carers were often responsible for convincing or even mildly forcing their relatives to give up some activities despite that such restrictions would be limiting and negatively affect their quality of life.

### Meeting the unpredictable healthcare system

#### Facing the inaccessibility of care

Carers sometimes experienced health care as unpredictable and inaccessible. When they sought hospital care for their relative, they received professional help but felt that genuine interactions with professional caregivers were rare. As carers, they felt that health care professionals were impersonal and sometimes demeaning. Moreover, they believed no one cared about their concerns and that many issues were left unattended.

[B]ut if you have COPD, you are admitted to the hospital and treated and then it’s bye-bye to you and then no one cares anymore. That is what I have experienced. (Informant 1)

Carers sought more contact with health care personnel and they wanted to receive information without repeatedly reminding the staff. The carers were also disconcerted because they do not receive timely and clear answers to their questions. One daughter wanted other resources so that her mother would not have to go to hospital every time her condition worsened, reasoning she could receive comparable care at home.

#### Being met with respect

The carers also had experiences of being well treated and recognized as individuals (e.g., when they had met someone within the healthcare system, they perceived was there for them and not focused on only treating the diagnosis.) They reported being taken seriously, listened to and receiving adequate help. These narratives often referred to a special person that the carer had met.

Now she also has an oxygen nurse […] she’s incredible and I can easily contact her. (Informant 9)

In addition, the carers referred to occasions when their contact with the healthcare sector resulted in a desirable care solution.

#### Feeling safe through shared responsibility

Carers felt safer when they shared some responsibility for their relatives with others (e.g., home care or home health care workers).

She lives by herself. So now, it is a safety for me that they (the home care services) comes that often. Because earlier I used to worry, if she did not answer the phone I wondered if she had deteriorated and maybe was unconscious or just asleep? So now, it feels safe to know that they check on her. (Informant.10)

This shared responsibility made their daily routine easier and reduced anxiety. Their concerns were partially alleviated if they knew a homecare worker’s responsibility included providing food for their relatives. The ability to depend on the homecare worker to make some decisions and ensure specific needs provided the carers with some relief.

### Comprehensive understanding

Our interpretation of the meaning of support for these carers, including our pre-understanding, the naïve reading and the structural analysis, is that it is a self-evident striving for the good life of their relatives and that they want to be *carers in partnership*. This interpretation is inspired by Ricoeur’s ethics on “aiming at the good life with and for others in just institutions” (Ricœur, 1994, p. 172). The support they provide their relatives is characterized by mutual respect and shared responsibility. In contrast, they are rarely involved as partners and co-creators of care in contact with health care professionals. Despite the formal (and sometimes impersonal) nature of healthcare institutions, a few carers could connect with a health care professional with whom they could share their frustrations and concerns. This connection was necessary to ensure that their relatives could maintain as good a life as possible, an outcome that should be the concern of a “just institution.” These kinds of open meetings between informal carers and formal caregivers were seen as positive and valued by the informants. We interpret these meetings as moments in which the carers have been included as equal partners in the care process.

## Discussion

In this study the carers did not explicitly explain their reasons for supporting their relatives. However, they viewed their support as a natural response or ethical position motivated by their love and compassion for their relatives. Referring to Aristotle’s *Nicomachean Ethics*, Ricoeur explains that the good life is found in praxis and that caring for others out of solicitude is an integral part of what constitutes a person’s self-esteem (Ricœur, 1994). For our informants, helping relatives was an obvious choice. To achieve this goal the carers supported their relatives in everyday life despite their deep frustrations with the healthcare system. Ateᶊ et al. described carers’ motives for caring to range from viewing caring as a duty to a natural response often motivated by love and compassion (Ates et al., 2018). The latter is similar to what we found in this study, i.e., the carers’ willingness to care (i.e., provide emotional, physical and instrumental support) for their relatives despite many difficult challenges.

Simpson et al. (2010) described caregiving as a duty for many carers and that they no longer saw the care recipient as the person they had once loved. Wives of men with COPD have reported that it is their responsibility to care for their husbands until the end of his life (Bergs, 2002). Also, in the present study, with exclusively female informants, supporting the relative was perceived as a self-evident responsibility, a natural condition of life. Strömberg (2013) suggests that women are more often expected to take on an informal carer role and female caregivers are more at risk of developing depression and anxiety. In addition, female caregivers show lower satisfaction with life than male caregivers (Yee & Schulz, 2000). Our findings confirm the view that supporting relatives is an unquestionable part of life but this fact has not been integrated in the health care system; informal caregivers often remain “outsiders”. We also noted that carers adjusted their lives to their relatives’ situation, including changing their plans to support the relatives in the best way. Similar findings have been reported by Simpson et al. (2010), Strang et al. (2018), and Petruzzo et al. (2017) and Wingham et al. (2017). These authors found that carers of persons with COPD or CHF adjust to the needs of their relatives in planning social events and everyday activities. In addition, carers of persons with CHF were always alert to potential acute events and determined to monitor their relatives for worsening symptoms (Brännström et al., 2007; Strøm et al., 2015). Not the least in these kind of situations but also in daily life caring it is important to be able to contact health care representatives for advice and support. In the present study such support was seldom offered, although desired. The carers see their respective relative as a person, not just a diagnosis, while health care is anonymous and often not reachable, viewing patients and carers more like objects than subjects. Even if the Swedish healthcare system performs relatively well, Scandinavian studies have found evidence of poor availability to care (e.g., Aasbo et al., 2017; Bove et al., 2016; Brännström et al., 2007; Strang et al., 2019; Westling, 2016). Carers struggle to participate in the health care process and are sometimes prevented from contributing with information or influencing decisions. When it comes to people with long-term health conditions, a great deal of the care and support that they need in their daily life is provided by informal carers and it would be hard for society to provide that type of care and support (Verbakel et al., 2017). Therefore, the healthcare system will have to incorporate these carers and join them in their effort to provide a good life for their relatives. To act as a support for their relatives the carers in this study expressed the need to have someone within the healthcare system that they could turn to and that would share some of the responsibility for their relative’s care.

Moreover, the comprehensive understanding of the present study underscores the need for a health care framework that acknowledges carers and patients as equal partners. This finding indicates that the traditional idea of viewing health care as the exclusive concern of experts needs to be transformed into a view that sees patients and their carers as active partners in care. Anker‐Hansen et al. (2018) advocate that a person-centred approach could help meet the often unmet support needs of informal carers. It underlines a holistic view of persons in which their relatives more easily can be included. Nygren Zotterman et al. (2018) contend that close relatives of people with a chronic illness want health care personnel to view them as part of the health care team. This finding is congruent with our comprehensive understanding.

In summary, our findings are supported by other studies, i.e., that health care is to some extent unpredictable and often inaccessible, which is remarkable considering that the sole existence of a healthcare system is to provide patients with high-quality care. Due to an ageing population, involvement of relatives in health care is expected to become increasingly important. Our findings indicate that there are relatives who are prepared to be involved as a resource in health care, but also emphasize that that there are relatives who are not given that opportunity. Current Swedish legislation requires that the patients’ relatives be included in the planning and implementation of their relatives’ care. On a European level, interest groups also request that carers be more involved in the health care process (Eurocarers, n.d.). Perhaps the accessibility of health care could also be increased if greater efforts were made to include carers as partners. To achieve this, the healthcare system will have to change its perspective in favour of a view that based on the patient´s preferences, resources and needs include the carer as an important partner in the planning and performing of care. An increased use of (already available) information and communication technology may be a feasible way to facilitate inclusion of carers in the patient´s everyday self-care activities, and in the interaction with healthcare (e.g., participate in care planning meetings digitally).

## Conclusion

Informal carers attempt to ensure a good life for their relatives and they want to be carers in partnership. In the future, the healthcare system must increase its efforts to involve informal carers by actively asking them about their situation and needs. Not least, the healthcare system must invite the carers to participate as partners in the evaluation and planning of care.

## Strengths and limitations

One major limitation of this study is that all the informants were women. However, women are more likely to be informal carers (Doherty et al., 2016; Verbakel et al., 2017).

The interviews were conducted by phone, which could be a weakness as it might be harder to capture nuances and details over the phone. It is hard to foresee how the interviews would be different if conducted in a face-to-face setting. On the other hand, the informants were recruited as relatives of the participants in another study (Fors et al., 2018) that evaluated the effect of person-centred phone support. Hence, interviewing by phone was in line with the methodology of that study, i.e., giving support by phone without face-to-face contact. An advantage of interviewing by phone was that it was easier to include informants who did not live nearby.

The interviews in this study were conducted by two persons, which could be seen as a limitation. However, the interviewers used the same opening questions as well as similar follow-up questions during the interviews. The first author, who had some earlier interviewing experience, conducted nine interviews, and the fourth author, who had extensive experience in interviewing, conducted five interviews.

Our informants were carers suggested by patients. All carers in this study cared for these patients on a regular basis. Because the carers were not randomly selected, we cannot infer that our results apply to a larger population of carers. Still, all the informants had the experience of being informal carers and were therefore able to share their unique experiences.

## Supplementary Material

Supplemental Material

## Data Availability

The participants of this study did not agree for their data to be shared publicly, so supporting data is not available.

## Notes

1. Chronic obstructive pulmonary disease.

2. Chronic heart failure.
