# Supplementary material for: Informal carers in Sweden – striving for partnership
Source: Int J Qual Stud Health Well-being. 2021 Oct 31;16(1):1994804. doi: 10.1080/17482631.2021.1994804 (PMC8567876; doi:10.1080/17482631.2021.1994804)
Supplement: Supplemental Material [file ZQHW_A_1994804_SM3772.zip › Supplementary files/Supplementary file 2.docx]

Supplementary file 2

Interview guide

This was not a guide that was followed point by point, after the initial questions we proceeded with the interview and tried to ask for explanations and more descriptions when needed.

- Could you tell me what support in your role as a carer means to you?
- Can you give me an example of when you were supported in caring for your relative?
- What do you think about when you think about support, can you describe that for me?
- Can you give me an example of when you have experienced good support for your relative?
- Can you give me an example of when you have experienced less good support for your relative?
- Could you tell me about what kind of support that you would wish for in your role as a carer?
